# Readiness of Health Care Professionals in Singapore to Teach Online and Their Technology-Related Teaching Needs: Quantitative Cross-sectional Pilot Study

**DOI:** 10.2196/42281

**Published:** 2023-03-06

**Authors:** Jason Wen Yau Lee, Fernando Bello

**Affiliations:** 1 Technology Enhanced Learning and Innovation Department Duke-NUS Medical School National University of Singapore Singapore Singapore; 2 Department of Surgery and Cancer Imperial College London London United Kingdom

**Keywords:** online readiness in teaching, technology for learning, faculty development, training need, technology-enhanced learning, readiness, teaching, medical education, health care education, teacher, teaching, online environment, online teaching, teaching skill, educator

## Abstract

**Background:**

With the increasing acceptance of face-to-face classes transitioning to web-based learning due to COVID-19, there is an increasing need to have educators trained and equipped to teach online. The ability to teach in-person may not necessarily mean that one is ready teach in a web-based environment.

**Objective:**

The objective of our study was to investigate the readiness of health care professionals in Singapore to teach online and their technology-related teaching needs.

**Methods:**

This was a quantitative cross-sectional pilot study conducted among health care administrative staff and professionals in medicine, nursing, allied health, and dentistry. Participants were recruited via an open invitation email to all staff members of Singapore’s largest group of health care institutions. Data were collected using a web-based questionnaire. Differences in the readiness of the professionals to teach online were analyzed using analysis of variance, and a 1-sided independent sample *t* test was performed to analyze the differences between respondents younger than 40 years and those older than 41 years.

**Results:**

A total of 169 responses was analyzed. Full-time academic faculty members scored the highest for readiness to teach online (2.97), followed by nursing professionals (2.91), medicine professionals (2.88), administrative staff members (2.83), and allied health professionals (2.76). However, there was no statistically significant difference (*P=.*77) among all the respondents in their readiness to teach online. There was an agreement among all professionals in their need for software tools to teach; in particular, there was a significant difference in the software needs among the professionals for streaming videos (*P*=.01). There was no statistically significant difference in the readiness to teach online between those younger than 40 years and those older than 41 years (*P*=.48).

**Conclusions:**

Our study shows that there are still some gaps in terms of readiness to teach online among health care professionals. Our findings can be used by policy makers and faculty developers to identify opportunities for development among their educators so that they are ready to teach online with the appropriate software tools.

## Introduction

### Background

By 2021, teaching online had become a norm in most institutions around the world because of the COVID-19 outbreak. The sudden change from in-person class sessions to web-based teaching platforms was accelerated with the need to socially distance and minimize face-to-face contact. Lecture halls and tutorial rooms that were once filled with students became empty, and classes were replaced with a monitor and a webcam during the COVID-19 pandemic. This caught many by surprise, and even full-time faculty members in academic institutions around the world were unprepared to teach online [1,2]. In Singapore, online teaching has been part of the national curriculum strategy since 2003 when SARS hit the country. Therefore, with the latest outbreak (COVID-19), educational activities for undergraduate and postgraduate continuous professional development [3] could be shifted online with little or no disruptions [4].

Although full-time faculty members may have received support from their institutions to teach online, it was evident that many medical faculty members did not receive adequate training on being effective educators even when they assumed major educational leadership roles in their institutions [5]. This could be attributed to the fact that there is a lack of recognition of the complex skills required for teaching [6], and most medical faculty members undergo ad hoc training after they assume their teaching roles [7]. This piecemeal approach to teaching and learning may not address the complex nature and needs of today’s learners and requires to be more structured. As such, various guides and teaching tips have been published over the past 2 years [8-10] to help educators transition to online teaching.

The Academic Medical Centre in Singapore recognizes the work of clinician-educators and places emphasis on faculty development across various professions. Their work has been ongoing with the establishment of the Academic Medicine Education Institute [11] in 2012 with the goal of providing faculty development training to the medical community across SingHealth [12]. The training programs are structured based on the Academy of Medical Educators (United Kingdom) professional standards framework [13]. However, the need to teach online prompted us to investigate the state of readiness among health care professionals to teach online within our health care academic institution. Although there has been a steady stream of research on online teaching and learning, there is a lack of agreement as to what constitutes the readiness of our educators to teach online. In this study, we developed a survey based on the existing literature to assess health care professionals’ readiness to teach online and their software-related needs for teaching online. We piloted the survey on the readiness to teach online across different health care professions and discuss our findings.

### Literature Review

#### Readiness to Teach and Learn Online

Online learning is becoming increasingly common, and there has been a growth in literature [2,14,15] examining learning in a web-based environment. Yet, one of the biggest challenges of teaching online is the tendency for educators to transfer traditional in-person teaching tenets into the web-based environment [16]. Such practices are usually the culmination of the educator’s past experience of emulating their own instructors that they consider as effective teaching [17] in a face-to-face environment. This is compounded by the fact that current circumstances forced many unprepared educators to change their teaching to a web-based environment. With little or no training prior to teaching online, educators will not only need to change their delivery approach but also learn how to use new technology-related tools.

Previous studies have argued that readiness to teach online can be conceptualized as the educator’s pedagogical [18-20] and mental preparedness [1,21] to develop and implement online teaching. A literature review by Cutri and Mena [19] found 5 major categories in past studies that conceptualized readiness to teach online: (1) educator’s belief and identity, which refers to the educator’s belief and identity when transitioning to a web-based course format; (2) transition to e-learning, which focuses on the transition process itself; (3) educator’s online competencies, which examines the educator’s skills in the online teaching format; (4) evaluation of online teaching and learning, which evaluates the educator’s ability to measure student learning outcomes; and (5) effectiveness of the teaching process, which reviews the educator’s teaching process.

#### Confidence and Familiarity With Teaching Online

The concept of self-efficacy represents the educator’s confidence in teaching [22] and refers to the measure of the educator’s ability to affect student success [23]. A comprehensive review of literature by Corry and Stella [24] showed that the educator’s self-efficacy in teaching online has a positive impact on student learning outcomes. They noted that the educator’s self-efficacy and technology integration was “especially important in online education since technology is central to both teaching and learning.”

Educators face a different set of challenges when teaching online compared to that in traditional face-to-face teaching settings. Apart from playing the role of a facilitator and content expert, educators will need to take on the role of a social administrator, technologist, counsellor, and researcher [25]. Fortunately, there is a myriad of learning tools available today for teaching and learning. For the novice educator, teaching online would not only mean juggling between content and pedagogy but also managing the technology and interaction surrounding the online teaching.

Studies [22,26] showing a strong correlation between the number of courses taught online and online teaching self-efficacy indicate that past experience in online learning has a positive impact on self-efficacy. What this means is that the more online experience that educators have in teaching, the higher is their confidence to do so. This finding was consistent with that reported in an earlier study back in 2007 by Lee and Tsai [27], who found that instructors with more web-related instructional experience had higher confidence in their classroom management ability. Therefore, the more the educators use technology to teach, the more they will be familiar with the technology.

#### Using Technology Effectively for Teaching and Learning

The nature of how learning takes place has changed with the increasing use of technology for teaching and learning. Learning can take place asynchronously, where interaction happens at the learner’s convenience, such as through discussion forums, e-learning modules, or video lectures. Synchronous learning aims to mimic traditional face-to-face learning where the learning takes place in real time, and learners log into a video conferencing system and interact with the educator in real time through audio, text-based chats, or various collaborative workspaces (eg, Google Docs, Miro). Online learning can be as effective as face-to-face learning [28], but the reality is that most educators are unprepared to transition from face-to face to online learning [2,29]. Being unprepared means that the educator would not effectively leverage the affordances of technology in their online classroom. In turn, the learning session would be a 1-way information delivery session with learners unable to interact with each other. Studies have reported that educators feel disconnected from their students in a virtual environment [28] since there is a loss of facial cues and teaching presence [30].

The way one would teach online is different from the way one would teach in a face-to-face session [17]. In asynchronous learning sessions, Coppola et al [31] suggested that technology can be used by educators not only to engage their learners in deeper cognitive activities but also on an affective level to develop deep intimate relationships with students. Traditional sets of teaching beliefs may be difficult to translate online, but online teaching opens new opportunities for educators to innovate and reflect on their teaching approaches that can be effectively enhanced by technology. Teaching is not just the delivery of content or transmission of information to students. Moreover, technology should not be used only as a means for content delivery or as a replacement for face-to-face contact.

#### Technology for Assessment

Constructive alignment [32] is a principle wherein teaching activities and assessment are aligned to the learning outcomes. Learning outcomes are clear, specific, and measurable statements that state the intention of the learning session or the module. When a course is constructively aligned, learning outcomes drive the teaching and learning activities, while assessments can be used to measure the extent learners achieved the outcome (summative assessment) or as feedback for improvement (formative assessment).

Educators need to re-examine the role that technology can play in assessments. For example, technology should not be limited to merely automate grading but rather to provide feedback to facilitate the development of reflective practice [33]. This can include using e-portfolios for learners to increase their sense of ownership across their various subject domains [34]. Studies have shown that technology can be effectively used for assessments such as peer evaluation with feedback, self-assessment, presentation, and online class participation [1,35,36].

## Methods

### Study Setting

This study was conducted with staff members of SingHealth, which is the largest group of public health care institutions in Singapore. SingHealth consists of 4 public hospitals, 3 community hospitals, 5 national specialty centers, and a network of 8 polyclinics.

### Sampling

All staff members who were experienced in teaching were included in this study, while those who did not have any experience in teaching were excluded. An invitation to participate in the web-based survey was sent through the SingHealth Corporate Communications Department to approximately 29,894 staff [37] members and was open for 5 weeks in March-April 2021. The staff members were from various professions such as medicine, nursing, allied health, dentistry, full-time faculty members, and administration. Prior to the start of the survey, respondents had the opportunity to read the participant information sheet and provide their consent electronically.

### Instrument

The survey was developed through an extensive literature review on similar studies [19-25], such as those measuring the readiness of educators to teach online. Based on the existing literature, we developed the items and conducted several revisions on the questions. To ensure face validity, we solicited feedback from 3 experts with in-depth knowledge of the medical education in Singapore. The survey was written in English, consisting of 4 items representing readiness to teach online, 5 items on technological tool needs, and 3 open-ended questions to understand the challenges faced when teaching online, the recommended technology tools, and other comments that the respondents may have. The survey used a 4-point Likert scale (4=strongly agree, 3=agree, 2=disagree, 1=strongly disagree) and a “not applicable” option if the statements did not apply to the respondents. Other demographic information collected included the profession, teaching frequency in the past 12 months, and age.

The survey to assess readiness to teach online consisted of 4 questions that measured (1) confidence in using technology for teaching, (2) familiarity with using technology for teaching, (3) ability to use technology effectively for teaching, and (4) ability to use technology tools to measure learner’s performance. The “readiness to teach online score” was calculated only for respondents who answered all the 4 questions in the survey.

The survey to assess the software tool needs for teaching and learning measured 5 types of software that could be used for (1) organizing their online teaching, (2) collaborative learning, (3) gaining insights into students’ learning progress, (4) promoting active learning, and (5) video streaming.

### Data Analysis

Statistical analysis was performed using SPSS for Mac version 27 (IBM Corp). To compare the mean scores across the various professions, means and standard deviations were calculated and analysis of variance (ANOVA) was used with a *P* value <.05 considered as statistically significant. To compare the mean scores across the 2 age groups, we conducted a 1-sided independent sample *t* test with a *P* value <.05 considered as statistically significant.

### Ethics Approval

This study was approved by the National University of Singapore’s Institutional Review Board (approval NUS-IRB-2020-437). This study was conducted following the Checklist for Reporting Results of Internet E-Surveys guidelines [38] (Multimedia Appendix 1).

## Results

### Overview

In this study, 331 responses were collected, with only 208 valid responses; 39 respondents indicated that they did not have any prior teaching experience and were excluded from the final analysis as they did not meet the inclusion criteria. Therefore, only 169 respondents were included in the final analysis.

### Characteristics of the Respondents

The largest number of responses was received from nursing professionals (n=65), followed by allied health professionals (n=40), medicine professionals (n=33), full-time academic faculty with no clinical appointment (n=9), administrative staff (n=8), and a small number from dentistry (n=3); 11 respondents did not state their profession. The respondents’ age groups differed across the professions, but 40.8% (69/169) of the respondents were aged 31-40 years followed by 29.6% (50/169) aged 41-50 years. Full-time faculty members were generally in their mid-to-late career, followed by the medical professionals. The age group profiles of the professionals in allied health, administration, and nursing were very similar, with many respondents in their early-to-mid career (31-40 years old). In terms of teaching frequency, 62.1% (105/169) of the respondents taught 1-5 times in the past 12 months, 15.4% (26/169) taught 6-10 times in the past 12 months, while 22.5% (38/169) taught more than 10 times in the past 12 months. More details on the respondents’ age groups and teaching frequency in the past 12 months across professions are shown in Table 1.

**Table 1:** Respondents’ age groups and teaching frequency in the past 12 months by profession.

		Administration (n=8), n (%)	Allied health (n=40), n (%)	Dentistry (n=3), n (%)	Faculty (n=9), n (%)	Medicine, (n=33), n (%)	Nursing (n=65), n (%)	Not known, (n=11), n (%)	Total (N=169), n (%)
**Age (years)**									
	20-30	1 (12.5)	6 (15)	0 (0)	0 (0)	0 (0)	7 (10.8)	0 (0)	14 (8.3)
	31-40	4 (50)	19 (47.5)	2 (66.7)	0 (0)	10 (30.3)	34 (52.3)	0 (0)	69 (40.8)
	41-50	1 (12.5)	14 (35)	0 (0)	2 (22.2)	14 (42.4)	19 (29.2)	0 (0)	50 (29.6)
	51-60	2 (25)	0 (0)	1 (33.3)	5 (55.6)	9 (27.3)	4 (6.2)	1 (9.1)	22 (13)
	61-70	0 (0)	1 (2.5)	0 (0)	2 (22.2)	0 (0)	1 (1.5)	0 (0)	4 (2.4)
	Unknown	0 (0)	0 (0)	0 (0)	0 (0)	0 (0)	0 (0)	10 (90.9)	10 (5.9)
**Teaching frequency (times in the last 12 months)**									
	1-5	4 (50)	28 (70)	2 (66.7)	5 (55.6)	10 (30.3)	47 (72.3)	9 (81.8)	105 (62.1)
	6-10	3 (27.5)	4 (10)	1 (33.3)	1 (11.1)	10 (30.3)	7 (10.8)	0 (0)	26 (15.4)
	>10	1 (12.5)	8 (20)	0 (0)	3 (33.3)	13 (39.4)	11 (16.9)	2 (18.2)	38 (22.5)

### Findings Across Professions

Table 2 shows the survey responses for readiness to teach online and software needs for teaching across health care professions. Respondents could select “not applicable” for any of the statements if it did not apply to them, and these responses were not included in the final tabulation. Therefore, the “n” for each item statement may be different. Dentistry was excluded from the analysis, as the sample size was too small (n<3) to make any meaningful conclusions.

Full-time academic faculty members scored the highest for readiness to teach online (2.97), followed by nursing professionals (2.91), medicine professionals (2.88), administrative staff members (2.83), and allied health professionals (2.76). A closer look at the survey on the readiness to teach online shows that full-time academic faculty members reported the highest agreement across the 3 statements of confidence in using technology for teaching, familiarity with using technology tools for teaching, and effectiveness in using technology for teaching, but they reported the lowest confidence in using technology for measuring learning outcomes. Respondents whose primary role was in administration reported agreement on statements relating to their confidence in teaching online and familiarity in using technology for teaching and learning but reported slight disagreement on their ability to use technology effectively in their teaching and for measuring learning.

**Table 2:** Survey responses for readiness to teach online and software requirements across different professions.

		Administration, n, mean (SD)	Allied Health, n, mean (SD)	Faculty, n, mean (SD)	Medicine, n, mean (SD)	Nursing, n, mean (SD)	*P* value
**Readiness to teach online**							
	I am confident in conducting classes online	8, 3.13 (0.64)	36,2.92 (0.77)	9, 3.22 (0.44)	33, 3.06 (0.79)	58, 2.95 (0.69)	.72
	I am familiar in using technology for teaching and learning	8, 3.0 (.076)	40, 2.84 (0.76)	9, 3.22 (0.44)	33, 3.0 (0.56)	62,2.84 (0.58)	.28
	I can use technology effectively in my teaching	8, 2.75 (0.71)	40, 2.65 (0.77)	9, 3.11 (0.6)	33, 2.85 (0.67)	63, 2.87 (0.63)	.33
	I can use technology-based tools to measure my learner's performance	7, 2.86 (0.69)	39, 2.64 (0.81)	9, 2.33 (0.87)	33, 2.61 (0.7)	61, 2.93 (0.6)	.05
	Overall readiness to teach online	7, 2.83 (0.66)	35, 2.76 (0.64)	9, 2.97 (0.48)	33, 2.88 (0.57)	57, 2.91 (0.5)	.77
**Software needs for teaching**							
	I require a software tool to organize my online teaching	8, 3.0 (0.87)	38, 3.08 (0.63)	9, 3.0 (0.87)	32, 2.91 (0.86)	59, 3.12 (0.53)	.69
	I require a virtual space for students to work together online	8, 3.0 (0.84)	37, 3 (0.8)	8, 3.37 (0.74)	30 2.9 (0.76)	59, 3.1 (0.48)	.43
	I require a software tool to gain insights into their learning progress	8, 3.13 (0.64)	36, 3.11 (0.7)	9, 3.44 (0.53)	31, 2.9 (0.83)	59, 3.19 (0.43)	.16
	I require tools to promote active learning	8, 3.38 (0.74)	38, 3.45 (0.65)	8, 3.63 (0.52)	32, 3.13 (0.71)	63, 3.3 (0.46)	.07
	I require a tool to record and stream videos to my students	8, 3.25 (0.7)	34, 3.29 (0.63)	9, 3.56 (0.73)	30, 2.83 (0.87)	59, 3.32 (0.47)	.01

Among the 3 health care profession groups, the medicine professionals reported agreement in their confidence in teaching online and familiarity with using technology tools for teaching. However, they reported mild agreement in their ability to use technology effectively for teaching and for measuring learning. For both nursing and allied health professionals, there was a mild agreement across all the 4 statements relating to their readiness to teach online. A 1-way ANOVA on the effect of profession on the readiness to teach online revealed only statistical significance in the ability to use technology to measure learner’s performance (*F*_4,144_=2.45; *P*=.05).

There was a universal agreement across professions that there was a need for software tools to promote active learning. In fact, most respondents across professions, except those in medicine, expressed a desire to have software tools to support their teaching. For respondents in the medicine profession, there was a slight disagreement on the need for tools for organizing their online teaching, collaborative learning, gaining insight into student learning progress, and video streaming (Table 2). One-way ANOVA was performed to analyze the effect of profession on the software needs for teaching. There was a significant difference between the software needs for video streaming (*F*_4,136_=3.81; *P*=.01) across professions.

### Findings Across Age Groups

Table 3 shows the survey responses on the readiness to teach online and the software needs for teaching across the age groups of 40 years or younger, and older than 40 years. There was almost an equal number of respondents when divided into these 2 age groups. The scores for readiness to teach online of those aged 40 years or younger (2.89), and of those older than 40 years (2.84) were very similar. A 1-sided independent sample *t* test on the readiness to teach online between respondents aged 40 years or younger, and those older than 40 years showed no significant difference across all the 5 items on the readiness. Similarly, there was no significant difference in the technology-related needs between respondents aged 40 years or below and those older than 40 years.

**Table 3:** Comparison of the survey responses across different age groups.

		Total, n, mean (SD)	≤40 years, n, mean (SD)	>40 years, n, mean (SD)	*P* value
**Readiness to teach online**					
	I am confident in conducting classes online	147, 2.99 (0.71)	74, 3.0 (0.68)	73, 2.97 (0.75)	.82
	I am familiar in using technology for teaching and learning	156, 2.9 (0.59)	82, 2.9 (0.6)	74, 2.89 (0.59)	.91
	I can use technology effectively in my teaching	156, 2.82 (0.68)	83, 2.84 (0.69)	73, 2.8 (0.67)	.65
	I can use technology-based tools to measure my learner's performance	152, 2.74 (0.71)	80, 2.83 (0.73)	72, 2.65 (0.7)	.14
	Overall readiness to teach online	151, 2.85 (0.55)	73, 2.89 (0.57)	71, 2.84 (0.53)	.48
**Software needs for teaching**					
	I require a software tool to organize my online teaching	149, 3.04 (0.68)	79, 3.06 (0.61)	70, 3.01 (0.75)	.66
	I require a virtual space for students to work together online	145, 3.05 (0.69)	76, 3.04 (0.64)	69, 3.06 (0.75)	.87
	I require a software tool to gain insights into their learning progress	146, 3.11 (0.66)	78, 3.1 (0.59)	68, 3.12 (0.72)	.89
	I require tools to promote active learning	152, 3.3 (0.63)	81, 3.33 (0.57)	71, 3.27 (0.7)	.52
	I require a tool to record and stream videos to my students	142, 3.2 (0.7)	74, 3.18 (0.63)	69, 3.22 (0.76)	.72

### Findings for Different Teaching Frequencies

Table 4 shows the mean readiness score and software needs for teaching based on the frequency of teaching in the past 12 months. There appears to be difference in the overall scores for readiness to teach online among those who taught 1-5 times (2.82), 6-10 times (2.71), and more than 10 times (3.01) in the past 12 months. In general, those who taught more often reported higher confidence in 4 of the dimensions of readiness to teach online. A 1-way ANOVA only showed statistical significance between familiarity using technology for teaching online (*F*_2,161_=4.89; *P*=.009) and the frequency of teaching in the past 12 months.

**Table 4:** Comparison of the survey responses based on the teaching frequency in the past 12 months.

		1-5 times, n, mean (SD)	6-10 times, n, mean (SD)	>10 times, n, mean (SD)	*P* value
**Readiness to teach online**					
	I am confident in conducting classes online	92, 2.89 (0.65)	26, 2.88 (0.82)	38, 3.13 (0.78)	.20
	I am familiar in using technology for teaching and learning	101, 2.78 (0.58)	26, 2.81 (0.69)	38, 3.13 (0.58)	.009
	I can use technology effectively in my teaching	100, 2.73 (0.68)	26, 2.69 (0.62)	38, 3.03 (0.68)	.05
	I can use technology-based tools to measure my learner's performance	97, 2.77 (0.72)	25, 2.52 (0.71)	38, 2.76 (0.71)	.28
	Overall readiness to teach online	88, 2.82 (0.53)	25, 2.71 (0.58)	38, 3.01 (0.56)	.07
**Software needs for teaching**					
	I require a software tool to organize my online teaching	95, 3.04 (0.62)	25, 2.92 (0.76)	37, 3.16 (0.73)	.37
	I require a virtual space for students to work together online	94, 3.05 (0.66)	22, 2.95 (0.72)	36, 3.11 (0.71)	.7
	I require a software tool to gain insights into their learning progress	95, 3.11 (0.66)	24, 3.0 (0.66)	35, 3.14 (0.73)	.72
	I require tools to promote active learning	100, 3.36 (0.56)	24, 3.25 (0.74)	36, 3.22 (0.72)	.46
	I require a tool to record and stream videos to my students	94, 3.27 (0.63)	22, 2.95 (0.65)	36, 3.14 (0.83)	.14

## Discussion

### Principal Findings

With face-to-face classes being kept to a minimum in Singapore since 2021, it is important to understand educators’ readiness to teach online and their requirements for software tools for conducting classes online. This survey was designed to be simple with only 9 items scored on a 4-point Likert scale to gauge health care professionals’ readiness to teach online and their needs for software tools to facilitate teaching online. The survey to assess readiness for teaching and learning online consisted of 4 questions developed based on existing literature on web-based learning and measures: (1) confidence in teaching online, (2) familiarity with technology tools, (3) effectiveness in using technology to teach, and (4) ability to use technology for measuring student learning. The survey on the technology-related needs for online teaching was administered to understand educators’ software needs for (1) organizing their online teaching, (2) collaborative learning, (3) gaining insights into students’ learning progress, (4) promoting active learning, and (5) video streaming.

When asked to rate their effectiveness to teach online, full-time faculty members rated themselves the highest (3.11); the rest of the health care professionals rated themselves below 2.97. Literature shows that it is common for health care educators to receive little or no training on how to become effective teachers [7,39] as compared with full-time faculty members and adjunct medicine faculty members who are likely to receive support from their respective medical schools. Nursing educators in SingHealth have a continuous education training program within their college but no dedicated teaching support resources available to them, which may explain their lower overall readiness to teach online. Allied health care professionals who responded to the survey comprised a diverse group of professionals (eg, radiologist, physiotherapist, pharmacist), which made identifying the gaps in the readiness to teach online challenging, as each profession has different needs, thereby making the training and teaching support more challenging.

Age alone does not appear to be a good determinant of one’s readiness to teach online. We found that there was almost no difference in the readiness to teach online between the younger and older cohorts of respondents. This finding was consistent with that reported by Eley et al [40] who found that nurses’ confidence to use technology was not determined by age alone but included a multitude of factors such as amount of exposure to the technology, frequency of technology usage, and workplace infrastructure. In addition to that, Singapore has a high digital literacy rate, especially among the working population [41] through various initiatives by the government under the SkillsFuture program [42], which may further explain why there was a lack of difference in the readiness to teach online between the 2 cohorts.

A study by Yeung et al [43] and Lee and Tsai [27] found that confidence to teach online was correlated with an educator’s teaching frequency. Indeed, our findings showed that those who taught very frequently in the past 12 months (>10 times) were more confident that than who taught less frequently. Respondents identifying as full-time faculty and medicine professionals who taught more frequently in the past 12 months had higher confidence in teaching online as compared with nursing and allied health professionals who did not teach so frequently. Therefore, the more one uses technology tools for teaching, the more confident they are with the affordances that these learning tools provide.

Assessment is an important part of teaching; yet, our findings showed that the ability to assess with technology was consistently rated low. A study by Schempp et al [44] on expert and novice teachers found that novice teachers often do not focus on assessments during their lesson planning as compared with their expert counterparts. We found that full-time faculty members and medicine professionals who were more experienced in teaching rated themselves the least confident in using technology for assessment, while nursing professionals who had lesser teaching experience rated themselves more confidently. Therefore, it is possible that the more experienced educators are aware of their inability to leverage technology for assessing their students, while novice educators may overestimate their confidence in using technology for assessments.

Thus, we found that one factor alone cannot be a strong determinant for readiness to teach online. We propose that a better way to understand an educator’s readiness to teach online is to consider multiple factors such as their teaching frequency, profession, and access to pedagogical resources. However, this would mean that we will require a higher response rate to make the findings more meaningful.

### Strengths and Limitations

Although we did not find statistically significant differences among health care professionals in their readiness to teach online or in their technology-related needs for teaching online, our findings are nonetheless important to be reported [45] and discussed. This first-of-its-kind study within our institution can be used to provide a snapshot of our educators’ readiness and software needs to teach online. We believe that our findings can be used to identify the training gaps that exist within our institution. One limitation of our study was that we did not collect data on the respondents’ previous faculty development training. It should not be assumed that anyone who has graduated from their respective field is capable of teaching [29,39]. For example, full-time faculty and medicine professionals would likely have more opportunities for faculty development training and more senior health care professionals will also likely have more opportunities over their career to attend faculty development training programs, which may explain their readiness to teach online. Due to the cross-sectional nature of our study, the second limitation of our study was that we were not able to establish causality beyond making assumptions on the findings. The sample size for the individual groups was too small to yield statistical significance and there was an unequal number of respondents across the professions. This could perhaps be attributed to the fact that the emails were sent by the corporate communications office and respondents were not compelled to complete the survey.

### Future Directions

The findings of our study are important to help identify training gaps in the corresponding educator training programs across different professions. Although faculty development training is conducted by the Academic Medicine Education Institute [11], our findings show that training opportunities should be targeted specifically at the different professions based on their needs. For example, allied health professional educators may require more targeted training so that their readiness to teach online can be on par with their nursing and medicine counterparts.

### Conclusion

Our study uses a 9-question survey to measure health care professionals’ readiness for teaching and learning (4 questions) online and their software needs for teaching and learning (5 questions). This survey was conducted in a health care setting in Singapore with various health care professionals. With online teaching and learning being here to stay for the foreseeable future, this survey will help institutions gauge the readiness of their educators to teach online. Findings from our survey can help future research, policy makers, and faculty developers allocate resources more effectively to address the gaps identified.

## Appendix

Multimedia Appendix 1 Checklist for Reporting Results of Internet E-Surveys (CHERRIES).

## Abbreviations

ANOVA: analysis of variance

## Data Availability

The data sets generated during and/or analyzed during the current study are available from the corresponding author on reasonable request.
