# Supplementary material for: Readiness of Health Care Professionals in Singapore to Teach Online and Their Technology-Related Teaching Needs: Quantitative Cross-sectional Pilot Study
Source: JMIR Med Educ. 2023 Mar 6;9:e42281. doi: 10.2196/42281 (PMC10028517; doi:10.2196/42281)
Supplement: Multimedia Appendix 1 [file mededu_v9i1e42281_app1.pdf]

Checklist for Reporting Results of Internet E-Surveys (CHERRIES)

Item Category	*Checklist Item*	*Explanation*	
**Design**			
	Describe survey design	Describe target population, sample frame. Is the sample a convenience sample? (In "open" surveys this is most likely.)	We wanted to target the survey to all our educators in SingHealth. To that, we used an open survey invite which was sent by the Corporate Communications office to all staff members in the organization
**IRB (Institutional Review Board) approval and informed consent process**			
	IRB approval	Mention whether the study has been approved by an IRB.	Ethics has been approved by the NUS Institutional Review Board (Approval Ref: NUS-IRB-2020-437)
	Informed consent	Describe the informed consent process. Where were the participants told the length of time of the survey, which data were stored and where and for how long, who the investigator was, and the purpose of the study?	Prior to the start of the survey, respondents had the opportunity to read the Participation Information Sheet and provided their consent electronically
	Data protection	If any personal information was collected or stored, describe what mechanisms were used to protect unauthorized access.	No identifiable information was collected
**Development and pre-testing**			
	Development and testing	State how the survey was developed, including whether the usability and technical functionality of the electronic questionnaire had been tested before fielding the questionnaire.	The survey was developed through an extensive literature review on similar studies such as those measuring the readiness of educators to teaching online. Based on the existing literature, we developed the items and conducted several revisions on the questions. To ensure face validity, we solicited feedback from 3 experts with in-depth knowledge of medical education in Singapore
**Recruitment process and description of the sample having access to the questionnaire**			
	Open survey versus closed survey	An "open survey" is a survey open for each visitor of a site,	The survey was open to all participants

Item Category	*Checklist Item*	*Explanation*	
		while a closed survey is only open to a sample which the investigator knows (password-protected survey).	
	Contact mode	Indicate whether or not the initial contact with the potential participants was made on the Internet. (Investigators may also send out questionnaires by mail and allow for Web-based data entry.)	Contact with participants were done online through email.
	Advertising the survey	How/where was the survey announced or advertised? Some examples are offline media (newspapers), or online (mailing lists – If yes, which ones?) or banner ads (Where were these banner ads posted and what did they look like?). It is important to know the wording of the announcement as it will heavily influence who chooses to participate. Ideally the survey announcement should be published as an appendix.	Recruitment was done through the Corporate Communication Office who sent out an open invitation via email to all SingHealth staff.
**Survey administration**			
	Web/E-mail	State the type of e-survey (eg, one posted on a Web site, or one sent out through e-mail). If it is an e-mail survey, were the responses entered manually into a database, or was there an automatic method for capturing responses?	Web survey
	Context	Describe the Web site (for mailing list/newsgroup) in which the survey was posted. What is the Web site about, who is visiting it, what are visitors normally looking for? Discuss to what degree the content of the Web site could pre-select the sample or influence the results. For example, a survey about vaccination on a anti-immunization Web site will have different results from a Web survey conducted on a government Web site	The survey was collected using survey platform called Qualtrics

Item Category	*Checklist Item*	*Explanation*	
	Mandatory/voluntary	Was it a mandatory survey to be filled in by every visitor who wanted to enter the Web site, or was it a voluntary survey?	Voluntary
	Incentives	Were any incentives offered (eg, monetary, prizes, or non-monetary incentives such as an offer to provide the survey results)?	Non incentives were offered
	Time/Date	In what timeframe were the data collected?	March – April 2021
	Randomization of items or questionnaires	To prevent biases items can be randomized or alternated.	No randomization
	Adaptive questioning	Use adaptive questioning (certain items, or only conditionally displayed based on responses to other items) to reduce number and complexity of the questions.	Adaptive questioning was applied. If respondents do not use certain technological tools, further questions on the tools will not be asked.
	Number of Items	What was the number of questionnaire items per page? The number of items is an important factor for the completion rate.	Between 1-5 questions
	Number of screens (pages)	Over how many pages was the questionnaire distributed? The number of items is an important factor for the completion rate.	6 pages
	Completeness check	It is technically possible to do consistency or completeness checks before the questionnaire is submitted. Was this done, and if "yes", how (usually JAVAScript)? An alternative is to check for completeness after the questionnaire has been submitted (and highlight mandatory items). If this has been done, it should be reported. All items should provide a non-response option such as "not applicable" or "rather not say", and selection of one response option should be enforced.	All questions displayed are required to be answered. If the items are not applicable to the respondents, they can select "Not applicable" as their response.

Item Category	*Checklist Item*	*Explanation*	
	Review step	State whether respondents were able to review and change their answers (eg, through a Back button or a Review step which displays a summary of the responses and asks the respondents if they are correct).	Respondents were able to use the Back button to review their answers but there was no summary of the responses
**Response rates**			
	Unique site visitor	If you provide view rates or participation rates, you need to define how you determined a unique visitor. There are different techniques available, based on IP addresses or cookies or both.	IP address and cookies were used
	View rate (Ratio of unique survey visitors/unique site visitors)	Requires counting unique visitors to the first page of the survey, divided by the number of unique site visitors (not page views!). It is not unusual to have view rates of less than 0.1 % if the survey is voluntary.	Not applicable
	Participation rate (Ratio of unique visitors who agreed to participate/unique first survey page visitors)	Count the unique number of people who filled in the first survey page (or agreed to participate, for example by checking a checkbox), divided by visitors who visit the first page of the survey (or the informed consents page, if present). This can also be called "recruitment" rate.	We are unable to determine the number of visitors who visited the first page of the survey as it was not captured by the system.
	Completion rate (Ratio of users who finished the survey/users who agreed to participate)	The number of people submitting the last questionnaire page, divided by the number of people who agreed to participate (or submitted the first survey page). This is only relevant if there is a separate "informed consent" page or if the survey goes over several pages. This is a measure for attrition. Note that "completion" can involve leaving questionnaire items blank. This is not a measure for how completely questionnaires were filled in. (If you need a measure for this, use the word "completeness rate".)	331 started the survey but only 208 completed the survey. Completion rate was 62%

Item Category	*Checklist Item*	*Explanation*	
**Preventing multiple entries from the same individual**			
	Cookies used	Indicate whether cookies were used to assign a unique user identifier to each client computer. If so, mention the page on which the cookie was set and read, and how long the cookie was valid. Were duplicate entries avoided by preventing users access to the survey twice; or were duplicate database entries having the same user ID eliminated before analysis? In the latter case, which entries were kept for analysis (eg, the first entry or the most recent)?	Cookies are automatically set by Qualtrics for each unique survey and prevent multiple submissions.<br><br>Cookie name: QST<br>Expires : 6 months<br><br>For more details on cookies, please see: https://www.qualtrics.com/support/survey-platform/getting-started/browser-cookies/
	IP check	Indicate whether the IP address of the client computer was used to identify potential duplicate entries from the same user. If so, mention the period of time for which no two entries from the same IP address were allowed (eg, 24 hours). Were duplicate entries avoided by preventing users with the same IP address access to the survey twice; or were duplicate database entries having the same IP address within a given period of time eliminated before analysis? If the latter, which entries were kept for analysis (eg, the first entry or the most recent)?	It is possible that multiple submission may come from the same IP address as respondents may be logged into same institution network.<br><br>Therefore, to prevent multiple submission, cookies are used.
	Log file analysis	Indicate whether other techniques to analyze the log file for identification of multiple entries were used. If so, please describe.	Not applicable
	Registration	In “closed” (non-open) surveys, users need to login first and it is easier to prevent duplicate entries from the same user. Describe how this was done. For example, was the survey never displayed a second time once the user had filled it in, or was the username stored together with the survey results and later eliminated? If the	Not applicable

Item Category	*Checklist Item*	*Explanation*	
		latter, which entries were kept for analysis (eg, the first entry or the most recent)?	
**Analysis**			
	Handling of incomplete questionnaires	Were only completed questionnaires analyzed? Were questionnaires which terminated early (where, for example, users did not go through all questionnaire pages) also analyzed?	All survey questionnaires that completed the Online Readiness to Teach part of the survey was included in the data analysis.
	Questionnaires submitted with an atypical timestamp	Some investigators may measure the time people needed to fill in a questionnaire and exclude questionnaires that were submitted too soon. Specify the timeframe that was used as a cut-off point, and describe how this point was determined.	Not applicable
	Statistical correction	Indicate whether any methods such as weighting of items or propensity scores have been used to adjust for the non-representative sample; if so, please describe the methods.	Not applicable
